# Effects of cattle manure and sludge vermicompost on nutrient dynamics and yield in strawberry cultivation with distinct continuous cropping histories in a greenhouse

**DOI:** 10.3389/fpls.2024.1514675

**Published:** 2025-01-06

**Authors:** Xiaofeng Bai, Wei Lu, Jin Xu, Qingyun Li, Zhanjun Xue, Xin-Xin Wang

**Affiliations:** College of Horticulture, Hebei Agricultural University, Baoding, China

**Keywords:** strawberry, vermicompost, yield and quality, substrate improvement, plant growth

## Abstract

Continuous cropping has emerged as a significant challenge affecting yield and quality in greenhouse strawberries, particularly as the cultivation of strawberries as a protected crop continues to increase. To address this issue, substrates with 0 or 2 years of continuous cropping were fertilized with two types of organic materials: vermicompost derived from either sludge or cattle manure. A control group consisted of substrate without the addition of vermicompost. Both type of vermicompost improved substrate fertility, promoted plant growth and fruit quality. The cattle manure vermicompost had a better improvement effect at peak fruiting stage. Substrate nutrients were increased 14.58~38.52% (0-year substrate) and 12.04%~42.54% (2-year substrate), respectively. In both substrate types, there was a substantial increase in microbial population and enzyme activity, accompanied by a significant decrease in phenolic acid content. During the senescence stage, the use of cattle manure vermicompost led to enhancements in plant height, leaf area, and root length, with increases ranging from 15.01% to 32.77% and 23.75% to 32.78% across the two substrate types compared to the control group. Furthermore, the application of cattle manure vermicompost significantly improved both fruit yield and quality. Compared with the control (CK), the cattle manure vermicompost increased fruit yield by 18.29% and 19.64% in the 0- and 2-year substrates, respectively. The contents of soluble sugars, vitamin C, and free amino acids in the fruits increased by 21.42%~34.16% (0-year substrate) and 9.62%~42.62% (2-year substrate), at peak fruiting stage. Cattle manure vermicompost application to the 2-year substrate ranked higher in the membership function than the CK treatment at 0-year planting. In conclusion, the application of vermicompost can significantly improve strawberry fruit yield and quality, as well as substrate characteristics, thus effectively addressing challenges associated with continuous cropping. Furthermore, the use of cattle manure vermicompost produced more pronounced positive effects.

## 1 Introduction

Strawberry (*Fragaria* × *ananassa* Duch.; family: Rosaceae genus: *Fragaria*) is an extremely adaptable perennial herb, extensively cultivated fruit globally. Strawberry renowned for its vibrant color, sweet flavor, and rich in vitamin C, antioxidants, minerals, and dietary fiber. It has substantial medicinal properties and anti-aging effects and is therefore designated “Queen of Fruits” (Yuan and Sun, 2022; Jiang et al., 2023). Strawberries are highly valued for their economic returns, making them a preferred crop among farmers, often leading to their continuous cultivation. However, this practice of continuous cropping has been shown to negatively impact both the quality and yield of strawberry plants (Dai et al., 2017).

It has been observed that continuous cultivation of the same crop or genetically similar crops in the same soil contributes to an increased accumulation of plant autotoxins and pathogenic microorganisms. This accumulation can lead to severe soilborne diseases and hindered plant growth, ultimately resulting in diminished crop yields (Hou et al., 2023). This phenomenon is commonly referred to as the continuous cropping obstacle (Cheng et al., 2023). The challenges associated with continuous cropping represent a widespread concern in the cultivation of greenhouse and facility plants, particularly in the case of strawberry production. Continuous cropping can indirectly influence the growth of strawberry plants by modifying soil chemistry, enzyme activities, and microbial community dynamics. These alterations can inhibit essential physiological processes, including photosynthesis and nutrient absorption. Consequently, the overall yield and quality of strawberries may be significantly compromised (Liu et al., 2024).

Organic remediation materials have emerged as effective solutions to help mitigate the challenges associated with continuous cropping, particularly in systems like strawberry cultivation (Wang et al., 2017a; Xu et al., 2021; Ma et al., 2023). By enhancing organic carbon sources and promoting soil microbial diversity, these materials can significantly improve soil quality. Research has shown that the application of organic remediation materials can lead to better nutrient availability, improved soil structure, and a healthier microbial environment (Niroshika et al., 2023).

Vermicompost is a natural bio-organic fertilizer that is characterized by its high content of organic matter and humic acids. It improves soil structure and permeability, enhances water infiltration and retention capacities, and elevates soil pH (Liu et al., 2022; Makkar et al., 2022). This effectively alleviates soil compaction issues in continuous cropping systems (Wu et al., 2019). Amylase, lipase, and cellulase present in vermicompost facilitate organic matter decomposition and nutrient cycling in the soil, thus boosting the activity of vital soil enzymes such as dehydrogenase, acid phosphatase, alkaline phosphatase, and urease (Liu et al., 2021). Furthermore, fresh vermicompost comprises a large population of beneficial microorganisms (Yang et al., 2015). Vermicompost can improve the number of soil microflora in greenhouses (Hou et al., 2023). It fosters the growth of soil bacterial and fungal populations, alters bacterial community structures, decreases the relative abundance of pathogenic fungi, and boosts the prevalence of beneficial microbial species, including *Bacillus* (Zhao et al., 2017; Wang et al., 2021a). Vermicompost application also improves nutrient content of the rhizosphere soil of *Atractylodes lancea* and augments microbial community abundance, thereby enhancing plant growth (Wang et al., 2021b). Vermicompost application was found to improve the agronomic traits of continuous cropping cucumbers, thus increasing their yield and quality (Feng et al., 2018). Vermicompost addition in cultivation soil mitigated continuous cropping issues and significantly augmented both yield and fruit quality (Wang et al., 2017b), Use of vermicompost in organic bag cultivation led to significant improvements in tomato plant growth compared to traditional soil cultivation methods, ultimately enhancing both fruit quality and yield (Jiang et al. (2022) These findings underscore the wide-ranging benefits of vermicompost for plant development. Renowned for its positive impact on plant nutrition and high bioavailability, vermicompost also helps diminish farmers’ dependence on chemical pesticides and inorganic fertilizers, promoting more sustainable agricultural practices (Nunes et al., 2018). However, studies on the application of vermicompost to alleviate continuous cropping challenges in strawberries, especially in greenhouse cropping substrates, remains limited.

This study utilized two types of vermicompost (derived from sludge and cow manure) in strawberry substrates cultivated for 0 and 2 years. This study posited two key hypotheses: (1) the two types of vermicompost enhanced the physicochemical and biological properties of the soil by increasing substrate pH, boosting organic matter content, balancing soil nutrient release, and regulating the soil microbial community, thereby alleviating the challenges associated with continuous cropping of strawberries; (2) these enhancements in substrate properties would subsequently lead to improved yield and quality of greenhouse strawberries, regardless of cultivation duration.

## 2 Materials and methods

### 2.1 Experimental materials and site

The experiment was conducted from mid of September to December 2023 in a greenhouse at the Hebei Shui run Jia he Agricultural Group Co., Ltd. base in Qingyuan County, Baoding City, Hebei Province (115°25′57″E, 38°44′19″N).

The substrates used for two planting durations (0-and 2- years) were sourced from Shui run Jia he in Qingyuan County, Baoding City (115°25′57″E, 38°44′19″N). The vermicompost used was collected from a vermicompost production base in Qingyuan County, Baoding City (115°28′12″E, 38°42′18″N). Two types of vermicompost were used: sludge vermicompost and cattle manure vermicompost, produced by decomposition of sewage sludge and cattle manure, respectively, by earthworms. Both types of vermicompost was crushed and sieved through a 2-mm mesh before applying to the substrate. The basic properties and heavy metal contents of the two vermicompost types are enlisted in **Supplementary Tables S1** and **S2**, respectively. Both vermicompost types comply with the GB 15618-2018 “Soil Environmental Quality Standards for Agricultural Land Soil Pollution Risk Control.” The ‘Xiangye’ strawberry variety was planted in the experiment.

### 2.2 Experimental design

The experiment employed a randomized complete block design with two factors. Two types of substrates, differentiated by the duration of continuous cropping (0 and 2 years), were subjected to three fertilization treatments: control (CK, local fertilization), sludge vermicompost (SV), and cattle manure vermicompost (CV). Each treatment was replicated four times, with a fully randomized arrangement within each block. Both types of vermicompost were applied at a 40% weight ratio as a one-time base fertilizer.

Strawberry seedlings, sourced from uniform conditions, were selected and planted in cultivation troughs measuring 1050 mm × 410 mm × 270 mm. Each treatment comprised eight cultivation troughs, ensuring a standardized approach to growth conditions across the various fertilization treatments. The plants were irrigated to achieve 100% field water-holding capacity using tap water, after which a uniform irrigation schedule was maintained for all treatments. Following acclimatization, the seedlings were covered with black plastic film mulch. Irrigation was managed via a drip system, with all plants drawing water from a common reservoir. Fertilization was administered through this system using a fully water-soluble fertilizer with a composition of N-P_2_O_5_-K_2_O: 20-20-20. The greenhouse conditions were controlled, maintaining daytime temperatures between 15~25°C and nighttime temperatures between 7~12°C, with relative humidity kept at 65%~75%. Effective management of weed growth and pest infestations was implemented throughout the experiment. Samples were collected and measured during the flowering, peak fruiting, and senescence periods.

### 2.3 Determination methods

#### 2.3.1 Physicochemical properties of the substrate

Physical properties were measured using the ring knife method according to Guo Shirong’s procedure (Wang et al., 2022). Chemical properties were determined using a Thunder Magnetic PHS-3C pH meter (INESA, Shanghai, China) and a DDS-307 conductivity meter (INESA, Shanghai, China), followed by saturated extraction method.

#### 2.3.2 Nutrient content of the substrate

After the substrates were dried, nutrient content was determined follow by Wang et al. (2021b). Total nitrogen was measured using the Kjeldahl method, total phosphorus was assessed via the NaOH alkaline fusion-molybdenum-antimony colorimetric method, and total potassium content was determined using the NaOH fusion method. Additionally, alkali-hydrolyzable nitrogen was quantified using the alkaline hydrolysis diffusion method, available phosphorus was extracted with sodium bicarbonate, effective potassium was measured using the ammonium acetate extraction method, and organic matter was analyzed through the potassium dichromate volumetric method.

#### 2.3.3 Substrate enzyme activity and microbial counts

After the substrates were air-dried indoors, they were ground and sieved through a 40-mesh. The activities of urease, polyphenol oxidase, peroxidase, sucrose enzyme, acidic phosphatase, and neutral phosphatase present in the substrate were measured using the corresponding enzyme activity test kits from Suzhou Grace Biotechnology Co., Ltd (Khan et al., 2024). Bacteria, fungi, and actinomycetes present in the substrate were cultured using the gradient dilution plate method (Liang et al., 2024). Beef extract peptone medium, Rose Bengal medium, and Gause I medium were used for bacterial, fungal, and actinomycetes cultures, respectively.

#### 2.3.4 Substrate phenolic acid substances

The phenolic acid content of the substrates in different treatment groups at the peak fruiting stage was determined through high-performance liquid chromatography (Wang et al., 2014).

#### 2.3.5 Plant growth indicators

Plant height was measured as the vertical height from the stem base to the highest point. The petiole length was measured as the distance from the leaf base to the tip of the petiole. Leaf area was measured by scanning the leaves with an AM200 leaf area meter.

#### 2.3.6 Plant chlorophyll content and photosynthetic indicators

Chlorophyll a, chlorophyll b, and carotenoids were extracted using the 80% acetone extraction method, and their contents were determined at wavelengths of 470, 645, and 663 nm, respectively (Jiang et al., 2016). A YZQ-100A photosynthesis meter (Yizong qi technology Co., Ltd, Beijing, China) was used to measure the photosynthesis rate, transpiration rate, intercellular CO_2_ concentration, and stomatal conductance of the functional strawberry leaves during the peak fruiting stage.

#### 2.3.7 Plant biomass

The plants were cut at the stem base and then washed, dried, and subjected to a preliminary drying at 105°C for 30 min in a forced-air oven. The samples were dried at 80°C for 48 h until a constant weight was attained. After that, the dry weight of each part was measured.

#### 2.3.8 Leaf physiological indicators and enzyme activity

Leaf physiological parameters were measured by Cao et al. (2007) method. Soluble sugar, soluble protein, and proline contents were measured using the anthrone method, Coomassie Brilliant Blue G-250 colorimetric method, and acid ninhydrin method, respectively. The malondialdehyde (MDA) content was measured using the thiobarbituric acid method. The activities of MDA, superoxide dismutase (SOD), peroxidase (POD), and catalase (CAT) were measured using enzyme activity assay kits (Suzhou Grace Biotechnology Co., Ltd.).

#### 2.3.9 Root physiological indicators

Root activity was measured using the triphenyl tetrazolium chloride method. The plant root systems were rinsed clean with tap water to remove any adhering substrate and other surface debris and then blotted dry. For each treatment, the root images were scanned using an MRS-9600TFU2L root scanner. Key parameters analyzed included root length, root surface area, root volume, and the number of root tips.

#### 2.3.10 Leaf nutrient content

After the strawberry leaves were dried, they were ground and passed through a 2-mm sieve. Nutrient content was analyzed following the methodology described by Wang et al. (2021b). Total nitrogen content was measured using the Kjeldahl method, total phosphorus content was assessed using the molybdenum-antimony colorimetric method, and total potassium content was determined.

#### 2.3.11 Fruit parameters

The single fruit weight was measured using an electronic balance. Horizontal and vertical fruit diameters were measured using a vernier caliper. The soluble solid content and free amino acid content were measured using a Japanese Atago PAL-1 digital refractometer and the ninhydrin colorimetric method, respectively (Cao et al., 2007). The ascorbic acid content and soluble sugar content were determined using a spectrophotometer and the anthrone-acetic acid ester method, respectively (Cao et al., 2007). The titratable acid content was determined using the NaOH standard solution titration method (Fan et al., 2023).

The fruit sugar–acid ratio was calculated as the ratio of the soluble sugar content to titratable acid content. The contents of the fruits’ sugar components (glucose, fructose, and sucrose) and acid components (citric acid and malic acid) were measured through liquid chromatography (Li et al., 2022). The yield per plant was recorded at harvest, and the total yield was calculated.

### 2.4 Data analysis

A three-factor analysis of variance (ANOVA) was employed to compare the effects of continuous cropping years, periods, and vermicompost treatments on the strawberry substrate, plants, and their fruits. Significance of differences was tested using one-way ANOVA (Duncan’s New Multiple Range Test) at *p* < 0.05 level. The data was presented as the arithmetic mean values with standard errors. The statistical analyses were performed with SPSS software, version 25.0 (IBM Corp., Armonk, NY, USA).

The data were analyzed using the fuzzy mathematics membership function method. The membership value for each component was calculated as follows:


$$\text{X}\left(\text{u}\right)=\left(\text{X}-{\text{X}}_{\text{min}}\right)/\left({\text{X}}_{\text{max}}-{\text{X}}_{\text{min}}\right)$$

If an indicator is negatively correlated, the inverse membership function is used for conversion. Calculate as follows:


$$\text{X}\left(\text{u}\right)=1-\left(\text{X}-{\text{X}}_{\text{min}}\right)/\left({\text{X}}_{\text{max}}-{\text{X}}_{\text{min}}\right)$$

An average of these values represented the mean membership value of the substrate properties during the peak fruiting period. Similarly, the mean membership values of the plant and fruit indicators were calculated. The structural equation modeling (SEM) analysis was performed on the SPSSPRO (https://www.spsspro.com/?utm_source=baidu-ss) platform to evaluate the direct and indirect effects of vermicompost addition on the substrate, plant growth, and fruit quality and yield. A hypothetical conceptual model including all reasonable pathways was considered (**Supplementary Figure S1**). All variables were standardized to improve normality in the residuals. The principal component analysis (PCA) was performed to simplify the variables under SEM, including “substrate properties,” “plant growth indicators,” “fruit morphological indicators,” and “fruit quality.” Specifically, “substrate properties” comprised substrate enzyme activity, microbial activity, and nutrient content and were represented by the PCA axis (PCA1). This explained 70.46% of the variance in substrate catalase activity, urease, sucrase, polyphenol oxidase, acid phosphatase, and neutral phosphatase activities. PCA1 represented “substrate microorganisms,” which explained 80.73% of the variance in bacteria, fungi, actinomycetes, and the bacteria-to-fungi ratio. “Substrate nutrients” were also represented by PCA1, which explained 74.34% of the variance in total nitrogen, total phosphorus, total potassium, alkali-hydrolyzable nitrogen, available phosphorus, available potassium, and organic matter contents (Liu et al., 2023). Pathways that did not contain relevant biological information were sequentially excluded.

## 3 Results

### 3.1 Responses of substrate, plant, and fruit traits to cropping years, period, and vermicompost treatments

The three-factor ANOVA indicated that vermicompost application significantly affected 51 traits, including substrate chemical properties and microbial counts (**Table 1**). Additionally, 49 traits each varied significantly with different time periods and continuous cropping durations. The interactions among cropping duration, time period, and vermicompost type affected more than half of the traits (*p* < 0.05).

**Table 1:** The results of three- way ANOVA [cropping years (Y), period (P), and treatment (T)] regarding four trait categories.

Categories	Traits	Cropping years (*Y*)	Period *(P*)	Treatment (*T*)	*Y*×*P*	*Y*×*T*	*P*×*T*	*Y*×*P*×*T*
Soil enzyme activities	Solid-Catalase activity (μmol/(h·g))	187.93 ***	822.46 ***	265.82 ***	39.67 ***	20.29 ***	4.97 **	7.72 ***
	Urease activity (μg/(d·g))	19.28 ***	55.61 ***	359.75 ***	2.05 ***	2.45 ***	1.48 ***	0.61 ***
	Sucrase activity (mg/(d·g))	290.28 ***	147.5 ***	108.01 ***	124.14 ***	6.39 **	7.59 ***	16.56 ***
	Polyphenol oxidase (μg/(d·g))	69.33 ***	38.27 ***	103.73 ***	5.06 *	7.50 **	1.01 ns	4.66 **
	Acid phosphatase activity (nmol/(h·g))	39.68 ***	56.41 ***	94.35 ***	7.11 **	11.92 ***	8.81 ***	7.32 ***
	Neutral phosphatase activity (nmol/(h·g))	145.64 ***	203.90 ***	126.25 ***	27.51 ***	1.17 ns	2.35 ns	0.79 ns
Soil cultivable microorganisms	Bacteria (10^7^cfu/g)	47.28 ***	16.04 ***	305.60 ***	0.81 ns	1.40 ns	2.66 *	3.01 *
	Fungi (10^4^cfu/g)	210.49 ***	125.80 ***	153.75 ***	7.82 **	1.59 ns	4.89 **	2.00 ns
	Actinomycetes (10^6^cfu/g)	45.75 ***	2.12 ns	193.64 ***	0.57 ns	1.83 ns	8.73 ***	0.51 ns
	Bacteria/Fungi	722.65 ***	223.88 ***	802.71 ***	114.65 ***	40.78 ***	9.83 ***	13.67 ***
Plant growth index	plant height (cm)	37.58 ***	98.19 ***	91.52 ***	3.72 *	5.38 **	5.03 **	4.43 **
	Petiole length (cm)	13.72 **	41.81 ***	13.04 ***	3.44 *	0.84 ns	0.18 ns	0.29 ns
	leaf area (mm^2^)	59.59 ***	2752.87 ***	57.02 ***	8.69 **	0.41 ns	4.11 **	0.08 ns
Plant biomass	Shoot dry weight (g)	99.62 ***	5.63 **	105.74 ***	14.37 ***	10.26 ***	7.67 ***	3.31 *
	Root dry weight (g)	72.80 ***	864.49 ***	88.86 ***	49.43 ***	3.23 *	20.68 ***	4.50 **
Chlorophyll content	Chlorophyll a (mg/g)	106.56 ***	131.12 ***	140.88 ***	5.43 **	2.34 ns	15.25 ***	3.44 *
	Chlorophyll b (mg/g)	29.35 ***	126.71 ***	81.84 ***	2.18 ns	2.72 ns	12.45 ***	2.64 *
	Carotenoids (mg/g)	68.89 ***	100.38 ***	38.01 ***	1.97 ns	5.96 **	11.58 ***	10.90 ***
Leaf enzyme activity	Superoxide dismutase activity (U/g)	6.26 *	99.14 ***	133.89 ***	1.86 ns	0.46 ns	6.45 ***	0.59 ns
	Peroxidase activity (ΔOD470/(min·g))	28.92 ***	172.10 ***	93.47 ***	0.81 ns	4.76 *	10.92 ***	1.97 ns
	Malondialdehyde content (nmol/g)	43.11 ***	1531.51 ***	91.90 ***	10.31 ***	11.42 ***	32.59 ***	8.10 ***
	Catalase activity (μmoL/(min·g)	401.98 ***	104.04 ***	107.07 ***	12.29 ***	3.86 *	7.80 ***	10.67 ***
Plant root physiological indicators	Root vitality (µgTTF/(g·h))	38.12 ***	1951.63 ***	107.60 ***	2.64 ns	5.52 **	21.54 ***	10.84 ***
	Root length (cm)	226.86 ***	1561.21 ***	83.90 ***	73.58 ***	4.16 *	13.59 ***	1.95 ns
	Root surface area (cm^2^)	72.63 ***	424.69 ***	31.42 ***	29.93 ***	0.38 ns	3.37 *	0.51 ns
	Root volume (cm^3^)	45.24 ***	428.42 ***	89.95 ***	1.77 ns	1.27 ns	3.60 *	1.57 ns
	Root mean diameter (mm)	72.10 ***	361.24 ***	40.47 ***	3.44 *	2.35 ns	4.10 **	0.78 ns
	Number of apex	21.63 ***	2436.99 ***	32.01 ***	2.11 ns	0.14 ns	7.75 ***	0.19 ns
	Root bifurcation number	262.36 ***	1617.78 ***	76.06 ***	115.47 ***	11.38 ***	9.70 ***	3.05 *
	Root crossing number	180.56 ***	1030.29 ***	50.04 ***	81.54 ***	0.24 ns	8.22 ***	0.53 ns
Fruit morphological index	weight of single fruit (g)	35.31 ***	157.04 ***	72.95 ***	3.00 ns	1.23 ns	5.58 **	0.05 ns
	Transverse diameter of fruit (mm)	5.07 *	65.58 ***	58.50 ***	0.09 ns	0.38 ns	9.20 **	0.93 ns
	Fruit longitudinal diameter (mm)	4.67 *	16.17 ***	45.93 ***	0.52 ns	0.58 ns	8.76 **	0.31 ns
Fruit quality index	soluble solids	59.86 ***	1.99 ns	187.80 ***	3.32 ns	2.10 ns	2.59 ns	0.63 ns
	Free amino acid content (mg/100g)	30.77 ***	265.91 ***	49.33 ***	0.94 ns	2.34 ns	11.38 ***	0.53 ns
	Vitamin C content (mg/100g)	26.80 ***	88.06 ***	46.74 ***	0.14 ns	2.20 ns	0.26 ns	2.10 ns
	Soluble sugar content (%)	64.60 ***	83.04 ***	70.67 ***	1.28 ns	6.37 **	4.57 *	0.73 ns
	Fructose content (mg/g)	34.86 ***	684.42 ***	94.49 ***	2.64 ns	0.68 ns	2.87 ns	3.28 *
	Glucose content (mg/g)	32.57 ***	413.23 ***	74.39 ***	17.73 ***	0.12 ns	0.24 ns	0.56 ns
	Sucrose content (mg/g)	2.83 ns	4024.00 ***	91.17 ***	1.21 ns	18.00 ***	31.62 ***	14.30 ***
	Titration acid content (%)	28.06 ***	20.71 ***	87.99 ***	18.08 ***	3.63 *	9.11 **	0.80 ns
	Citric acid content (mg/g)	30.29 ***	2.51 ns	40.78 ***	24.93 ***	0.99 ns	1.78 ns	6.50 **
	Malic acid content (mg/g)	97.83 ***	116.61 ***	56.13 ***	1.39 ns	1.42 ns	0.70 ns	5.84 **
	Sugar-acid ratio	95.07 ***	147.76 ***	220.41 ***	10.10 **	2.87 ns	20.59 ***	2.26 ns

The values in the table are F values. ns, not significant; *0.01 ≤ *p* < 0.05; **0.001 ≤ *p* < 0.01; ***p < 0.001.

### 3.2 Substrate properties

#### 3.2.1 Chemical properties, nutrient content, and phenolic acid content of substrates

**Figure 1:**
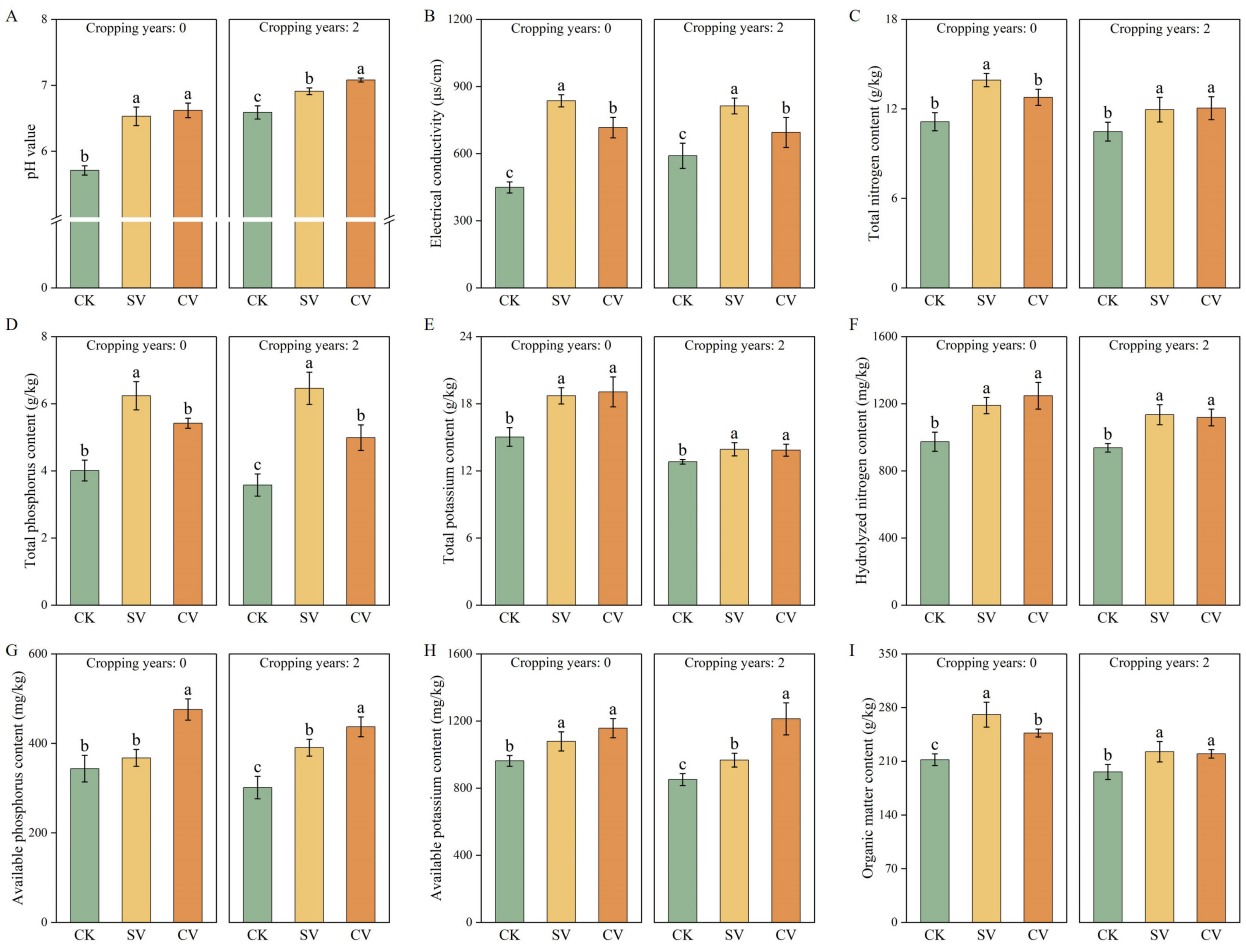
Effect of vermicompost on pH value **(A)**, electrical conductivity **(B)**, total nitrogen content **(C)**, total phosphorus content **(D)**, total potassium content **(E)**, hydrolyzed nitrogen content **(F)**, available phosphorus content **(G)**, available potassium content **(H)** and organic matter content **(I)** at the peak fruiting period. The letters indicate the significant difference of the influence of different vermicompost on the index, and the significance level is *p* < 0.05. Abbreviations for treatment: Control (CK), sludge vermicompost (SV) and cattle manure vermicompost (CV).

**Figure 2:**
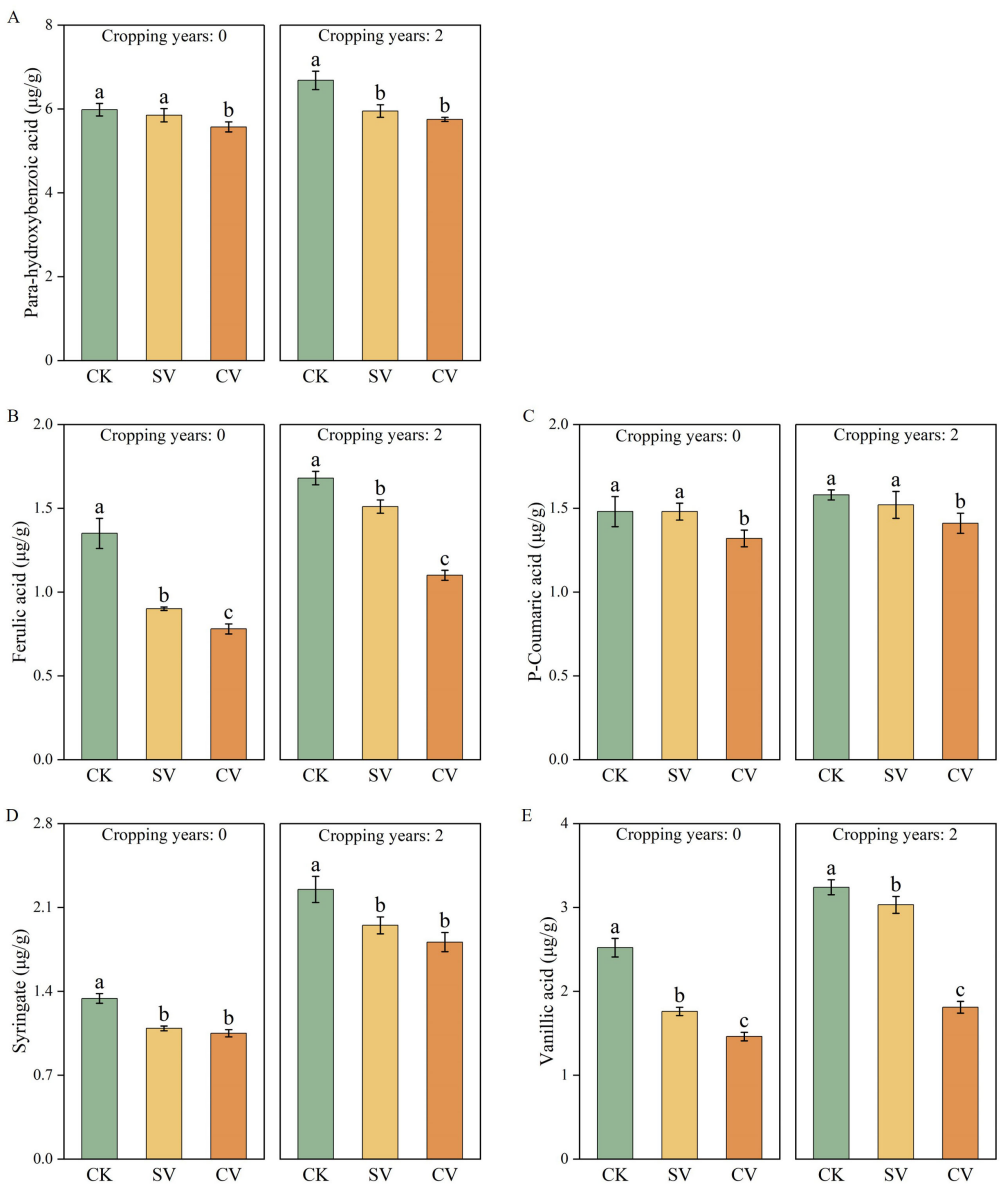
Effects of vermicompost on para-hydroxybenzoic acid **(A)**, ferulic acid **(B)**, p-coumaric acid **(C)**, syringate **(D)** and vanillic acid **(E)** during the peak fruiting period. The letters indicate the significant difference of the influence of different vermicompost on the index, and the significance level is *p* < 0.05. Abbreviations for treatment: Control (CK), sludge vermicompost (SV) and cattle manure vermicompost (CV).

In both substrate types, vermicompost treatments significantly increased pH and electrical conductivity (EC) values (**Figures 1A, B**). In the 0-year substrate, pH and EC increased by 6.62%~14.32% and 59.52%~86.25%, respectively, compared to the control. In the 2-year substrate, pH and EC rose by 6.91%~7.08% and 17.65%~37.68%, respectively, relative to the control. Vermicompost treatments significantly enhanced substrate nutrient content (**Figures 1C–I**), with total phosphorus and available phosphorus showing the most pronounced increases (*p* < 0.05). The SV treatment notably increased total phosphorus content, while the CV treatment significantly enhanced available phosphorus (*p* < 0.05). Moreover, vermicompost application significantly reduced phenolic acid content (**Figure 2**), particularly ferulic and vanillic acids, which showed the greatest declines. In the 0- and 2-year substrates, the CV treatment led to reductions in ferulic acid levels by 42.54% and 34.69% compared to SV and CK treatments, respectively; vanillic acid levels were reduced by 42.57% and 44.36%, respectively.

#### 3.2.2 Substrate enzyme activity and cultivable microorganisms

**Figure 3:**
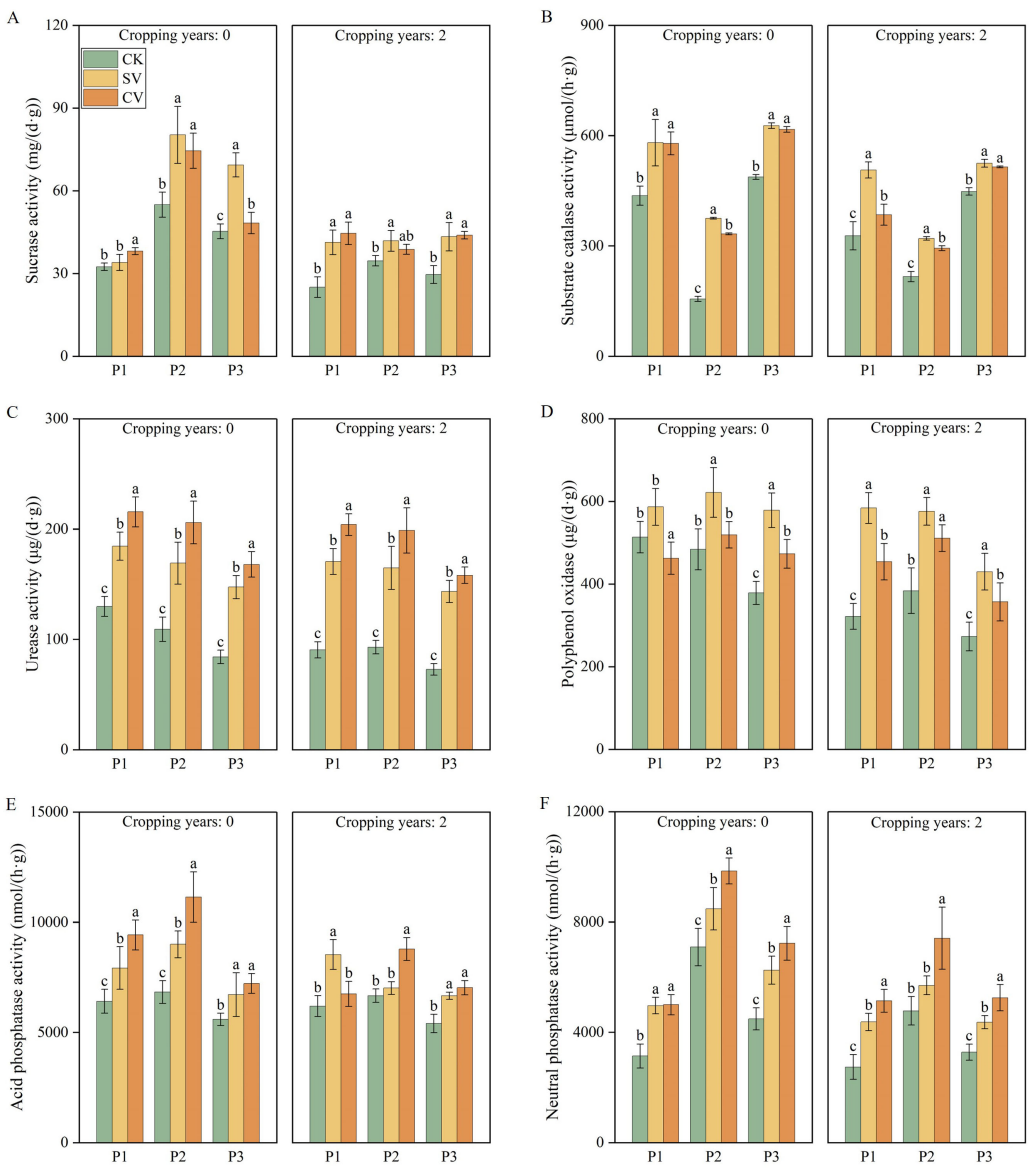
Effects of vermicompost on sucrase activity **(A)**, substrate catalase activity **(B)**, urease activity **(C)**, polyphenol oxidase **(D)**, acid phosphatase activity **(E)** and neutral phosphatase activity **(F)** in different periods. Letters indicate significant differences in the effects of different vermicompost treatments on this indicator at different time periods, with a significance level of *p* < 0.05. P1, P2 and P3 represent flowering period, peak fruiting period and the senescence period respectively. Abbreviations for treatment: Control (CK), sludge vermicompost (SV) and cattle manure vermicompost (CV).

**Figure 4:**
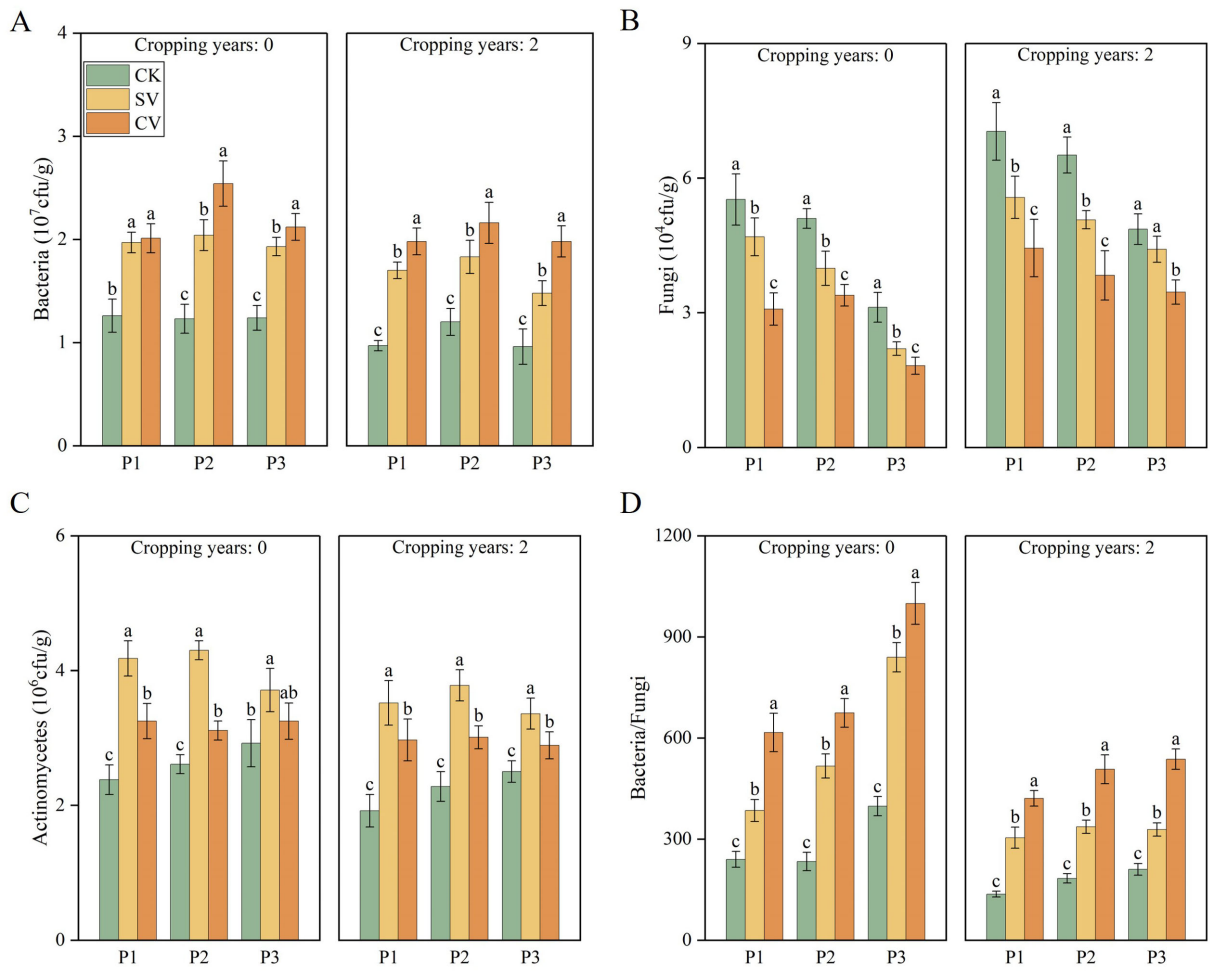
Effects of vermicompost on bacteria **(A)**, fungi **(B)**, actinomycetes **(C)** and bacteria/fungi **(D)** in different periods. Letters indicate significant differences in the effects of different vermicompost treatments on this indicator at different time periods, with a significance level of *p* < 0.05. P1, P2 and P3 represent flowering period, peak fruiting period and the senescence period respectively. Abbreviations for treatment: Control (CK), sludge vermicompost (SV) and cattle manure vermicompost (CV).

The two vermicompost treatments significantly increased the enzymatic activities and microbial indices in the two substrates (**Figures 3**, **4**). Catalase and polyphenol oxidase exerted significant effects in the SV treatment group compared with the CK group (*p* < 0.05), with their activities increasing by 140.83% and 47.86%, respectively, at fruit stage. Urease, acid phosphatase, and neutral phosphatase activities were notably higher in the CV treatment group, with urease activity increasing by 113.92% and 88.60% compared to CK at the full fruit stage in the 0- and 2-year substrates, respectively (*p* < 0.05). Different vermicompost treatments significantly enhanced bacterial and actinomycete populations and the bacterial-to-fungal ratio in the substrates while significantly reducing fungal counts (*p* < 0.05). The SV treatment group exhibited the most significant effects on actinomycetes, whereas the CV treatment group had the greatest impact on other bacterial and fungal populations and the bacterial-to-fungal ratio. In substrates with 0- and 2-year planting histories, the bacterial-to-fungal ratio under the CV treatment was highest, increasing by 188.89% and 176.46%, respectively, at the full fruit stage compared to the CK treatment. These results suggest that cattle manure vermicompost enhances substrate resistance to fungal diseases.

### 3.3 Plant characteristics

#### 3.3.1 Plant growth indicators

**Figure 5:**
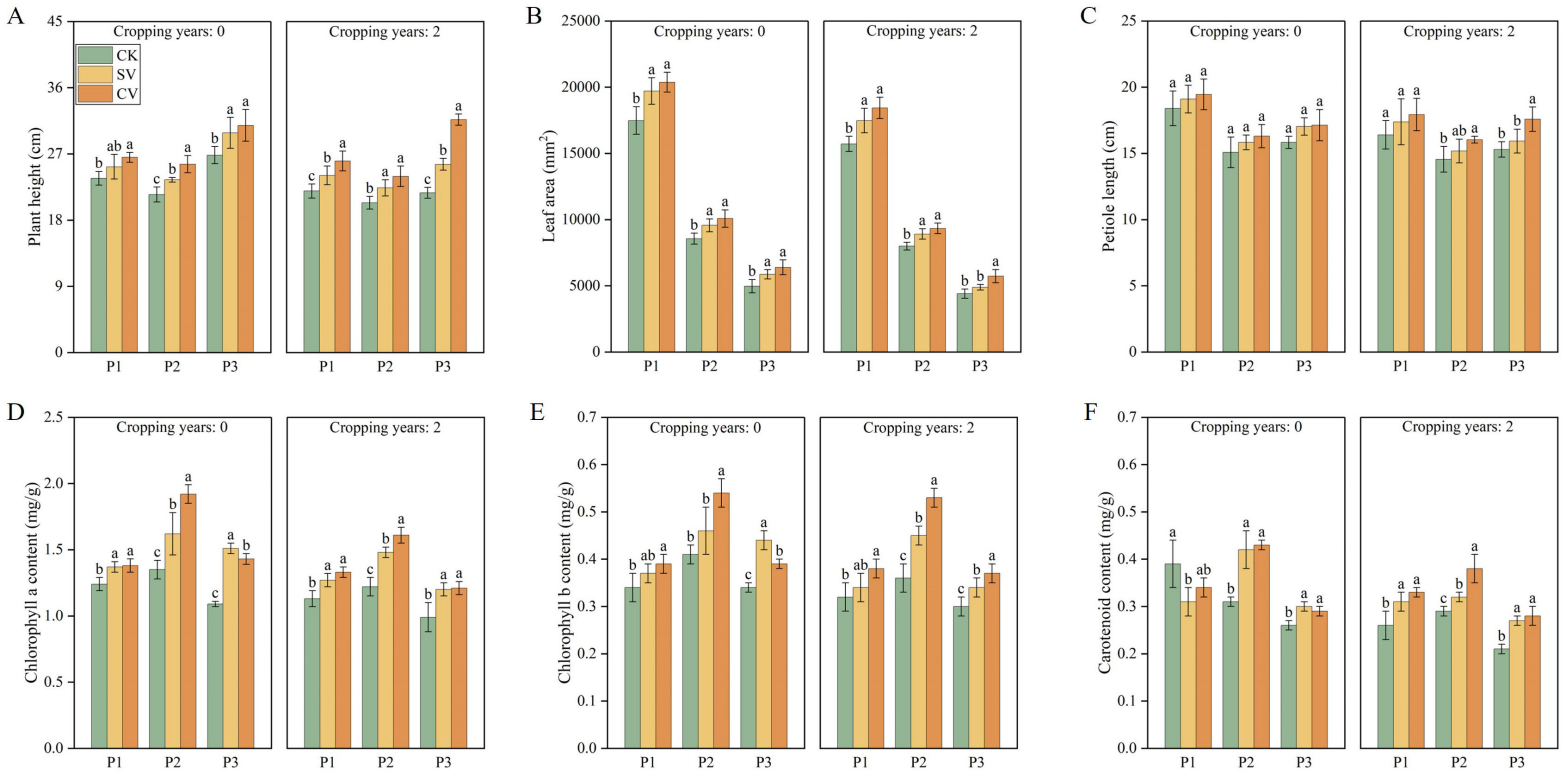
Effects of vermicompost on plant height **(A)**, leaf area **(B)**, petiole length **(C)**, chlorophyll a content **(D)**, chlorophyll b content **(E)**, carotenoid content **(F)** in different periods. Letters indicate significant differences in the effects of different vermicompost treatments on this indicator at different time periods, with a significance level of *p* < 0.05. P1, P2 and P3 represent flowering period, peak fruiting period and the senescence period respectively. Abbreviations for treatment: Control (CK), sludge vermicompost (SV) and cattle manure vermicompost (CV).

In the substrates without and with 2-year cropping history, the vermicompost treatments profoundly affected plant height, leaf area, chlorophyll content, photosynthetic parameters, and leaf nutrients at different growth stages (**Figures 5**; **Supplementary Figures S1**, **S2**). In the 0- and 2-year substrates, the CV treatment significantly increased plant height and leaf area, especially during aging (*p* < 0.05). The plant height in the two substrates increased by 15.10% and 23.75%, and the leaf area increased by 28.71% and 30.02%, respectively, compared with the CK treatment. The chlorophyll b content in the CV treatment group exhibited significant increase of 32.80% and 47.91% compared with the CK treatment at fruit stage. Moreover, vermicompost treatments significantly increased the photosynthesis rate, transpiration rate, and stomatal conductance of the leaves but reduced the intercellular CO_2_ concentration. In the 0-year substrate, the photosynthetic and transpiration rates following the CV treatment increased by 15.53% and 24.35% compared with the CK treatment, respectively. In the 2-year substrate, the photosynthesis rate following the CV treatment increased by 41.60% compared with the CK treatment. Vermicompost application significantly increased the nutrient content of the leaves, particularly the total nitrogen content (*p* < 0.05). In the 0-year and 2-year substrates, total nitrogen content was elevated by 31.73% and 41.62%, respectively, following the CV treatment compared to the CK treatment. Additionally, the CV treatment significantly increased total phosphorus and total potassium contents.

#### 3.3.2 Plant biomass and leaf enzyme activity

**Figure 6:**
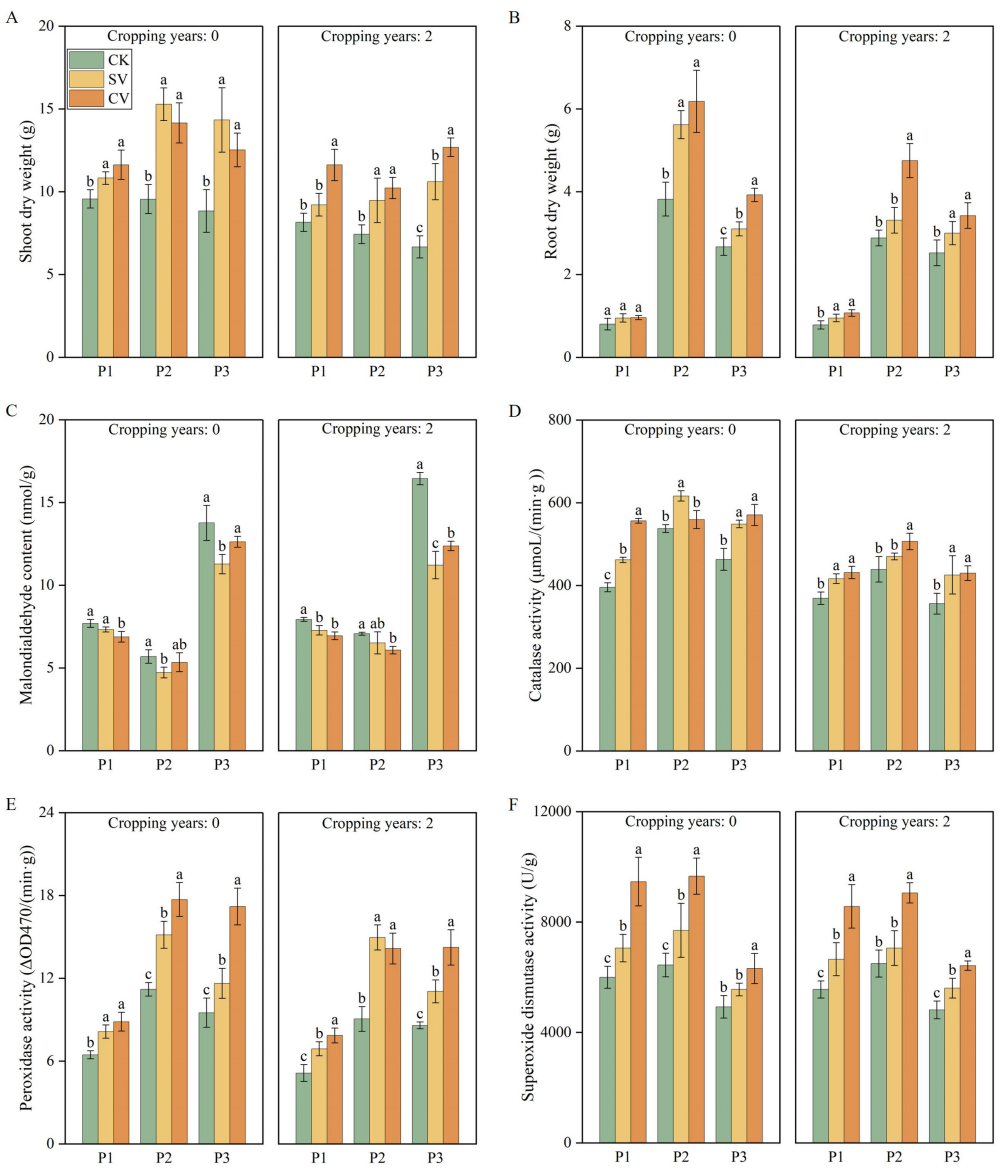
Effects of vermicompost on shoot dry weight **(A)**, root dry weight **(B)**, malondialdehyde content **(C)**, catalase activity **(D)**, peroxidase activity **(E)** and superoxide dismutase activity **(F)** in different periods.. Letters indicate significant differences in the effects of different vermicompost treatments on this indicator at different time periods, with a significance level of *p* < 0.05. P1, P2 and P3 represent flowering period, peak fruiting period and the senescence period respectively. Abbreviations for treatment: Control (CK), sludge vermicompost (SV) and cattle manure vermicompost (CV).

Cropping years, periods, vermicompost treatments, and the interaction between periods and vermicompost treatments were found to have significant effects on plant biomass and leaf enzyme activities (**Table 1**; **Figure 6**). The vermicompost treatments significantly affected aboveground and belowground dry weights, and leaf enzyme activities (*p* < 0.05). In the 0-year substrate, the SV treatment resulted in highest increase of 62.31% in the aboveground dry weight during the aging period, increase compared with the CK treatment. In the 2-year substrate, the CV treatment demonstrated significant increase (90.46%) in the aboveground dry weight during the aging period. In both substrates, the CV treatment led to the most significant increase in the belowground dry weight at the fruit stage, with increases of 61.54% and 64.70% compared with the CK treatment, respectively. The vermicompost treatments significantly reduced leaf MDA levels and notably increased CAT, POD, and SOD activities (*p* < 0.05). The most substantial changes in MDA levels were observed during the aging period; specifically, the CV treatment led to an 18.13% reduction in MDA levels in the 0-year substrate, while the SV treatment resulted in a 31.85% decrease in the 2-year substrate. Regarding POD activity, the CV treatment had the most pronounced effect during the aging period in both substrates, enhancing POD activity by 81.05% and 65.89%, respectively, compared to the CK treatment (*p* < 0.05).

#### 3.3.3 Plant root physiological indicators

Cropping years, periods, and vermicompost treatments, and two-factor interactions between periods and vermicompost treatments significantly affected the eight root physiological indicators of the plants (**Table 1**; **Supplementary Figure S4**). Adding vermicompost to both substrate types significantly increased root vitality, promoting the growth of strawberry plant roots (*p* < 0.05). The most pronounced changes in root vitality occurred at the peak fruiting stage in both substrates. The CV treatment significantly enhanced root vitality in both substrates, showing increases of 19.47% and 68.72%, respectively, compared to the CK treatment (**Supplementary Figure S4A**). Among the various root growth indicators, root volume exhibited the most significant response to the SV treatment across the different substrates with varying planting histories (*p* < 0.05). While no significant differences were noted between the SV and CV treatments for other root growth indicators, both treatments demonstrated effects that were significantly greater than those of the CK treatment.

### 3.4 Effect of vermicompost treatments on strawberry yield and quality

**Figure 7:**
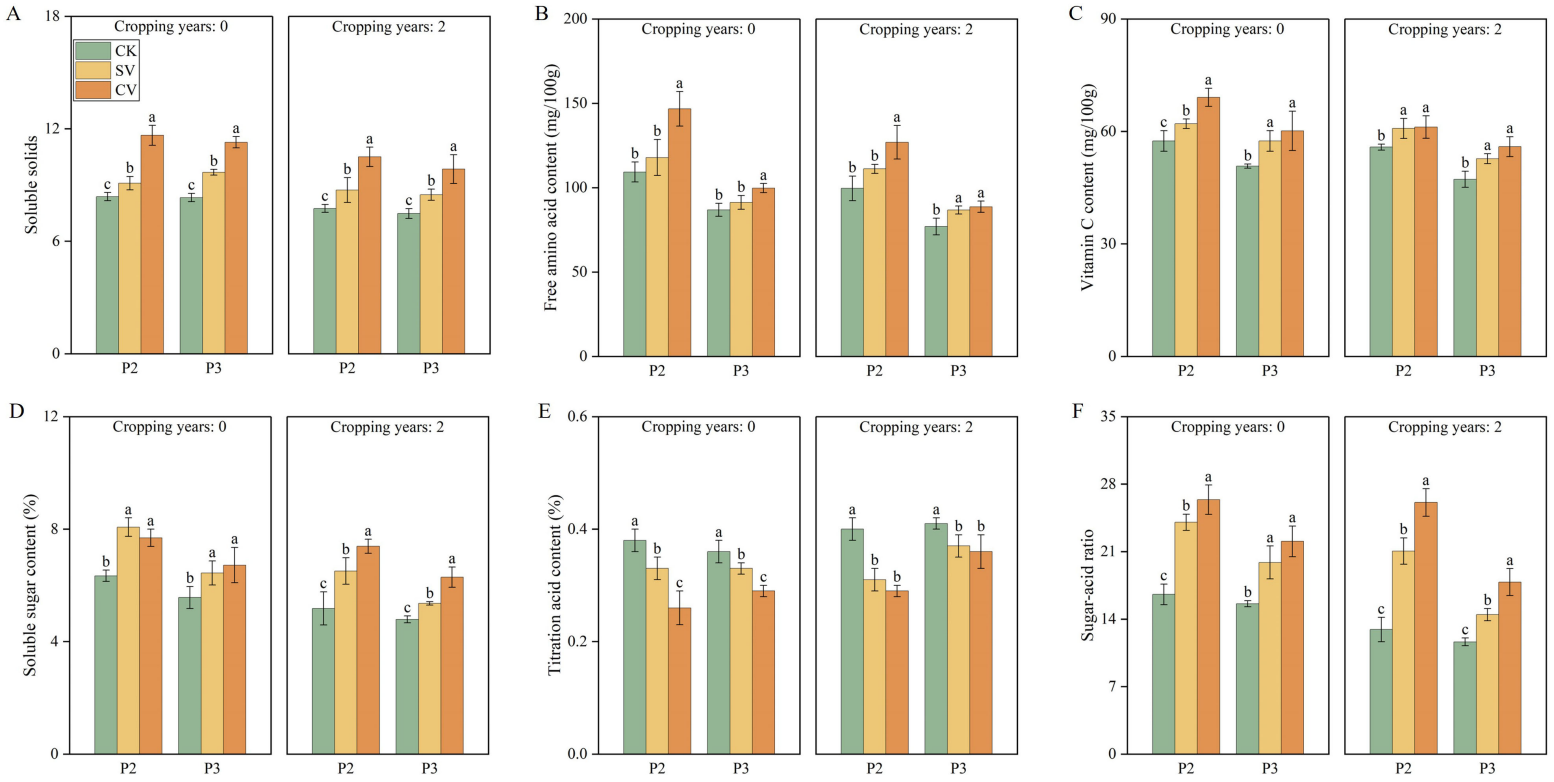
Effects of vermicompost on soluble solids **(A)**, free amino acid content **(B)**, vitamin c content **(C)**, soluble sugar content **(D)**, titration acid content **(E)** and sugar-acid ratio **(F)** in different periods. Letters indicate significant differences in the effects of different vermicompost treatments on this indicator at different time periods, with a significance level of *p* < 0.05. P2 and P3 represent peak fruiting period and the senescence phase respectively. Abbreviations for treatment: Control (CK), sludge vermicompost (SV) and cattle manure vermicompost (CV).

Cropping years, periods, and vermicompost treatments significantly affected the morphology, yield, and quality of strawberry fruits (**Table 2**). In both planting substrates, the vermicompost treatment significantly increased fruit weight, diameter, and yield, with prominent effects particularly observed at the peak fruiting period (*p* < 0.05). The CV treatment significantly increased both fruit weight and diameter by 18.29% and 19.64%, respectively compared with the CK treatment. Continuous cropping significantly reduced fruit quality (**Table 1**; **Figures 7**; **Supplementary Figure S5**). The addition of vermicompost to the 0- and 2-year substrates significantly enhanced the levels of soluble solids, sugars, free amino acids, and vitamin C, while also increasing the sugar-to-acid ratio and decreasing titratable acidity (*p* < 0.05). Notably, the CV treatment produced the most significant effects across all stages, achieving the highest sugar-to-acid ratios of 26.38 and 26.10 for the 0-year and 2-year substrates, respectively, at the peak fruiting period.

**Table 2:** Analysis of the fuzzy mathematical membership functions for component content.

Cropping years	Treatment	Mean of membership function				Ranking
		Substrate properties	Plant indicators	Fruit indicators	Total components	
0	CK	0.31	0.32	0.36	0.33	5
	SV	0.80	0.75	0.68	0.76	2
	CV	0.88	0.89	0.76	0.86	1
2	CK	0.01	0.00	0.20	0.05	6
	SV	0.46	0.35	0.51	0.43	4
	CV	0.59	0.62	0.64	0.62	3

### 3.5 Impact of cattle manure vermicompost on strawberry fruit yield and quality in different cropping histories

The effects of the cattle manure vermicompost on fruit yield and quality varied between the substrates without and with two years of continuous cropping (**Table 3**). The increases in the fruit soluble sugar content and sugar–acid ratio was the highest in the 2-year substrate, differing significantly from those in the 0-year substrate (*p* < 0.05). The increase in the fruit vitamin C content and decrease in the titratable acid content (%) were the highest in the 0-year substrate.

**Table 3:** Increased or decreased percentage in fruit yield or fruit quality in CV treatment compared with CK on each substrate.

Cropping years	Yield (%)	Soluble sugar content (%)	Titration acid content (%)	Sugar-acid ratio (%)	Free amino acid content (%)	Vc (%)
0	18.25 ± 2.33 a	21.43 ± 3.44 b	-32.26 ± 4.89 a	59.27 ± 3.51 b	34.31 ± 7.81 a	20.32 ± 3.20 a
2	19.71 ± 3.10 a	43.67 ± 12.45 a	-29.26 ± 1.89 a	102.81 ± 10.73 a	27.47 ± 5.82 a	9.59 ± 4.23 b

“(CV – CK)/CK * 100” was used here. Means (± SE) followed by different letters denote significant differences according to Tukey test (*p* < 0.05) among two substrate treatments under each column.

### 3.6 Correlation between substrate parameters, plant growth, fruit yield, and quality

**Figure 8:**
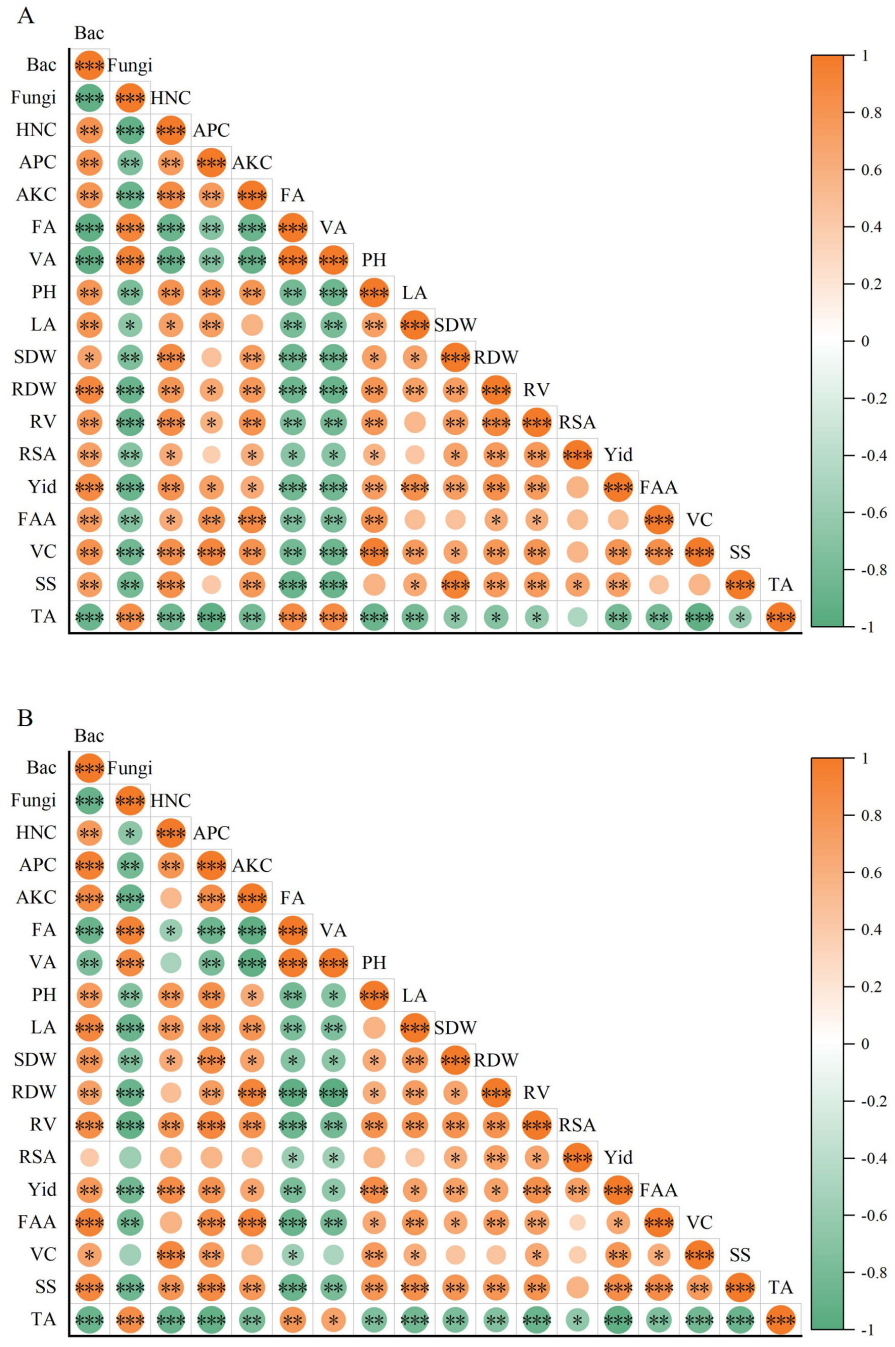
Correlation plot between substrate parameters, plant growth, fruit yield, and quality under different vermicast treatments. **(A)** and **(B)** represent substrates with 0 years and 2 years of planting history, respectively. Red and green circles indicate positive (0 ≤ *p* ≤ 1) and negative (-1 ≤ *p* < 0) correlations. *0.01 ≤ *p* < 0.05; **0.001 ≤ *p* < 0.01; ***p < 0.001. The diameter and color intensity of the circles are proportional to the magnitude of the Pearson correlation coefficient. Bac, Bacteria; Fungi, Fungi; HNC, Hydrolyzed nitrogen content; APC, Available phosphorus content; AKC, Available potassium content; FA, Ferulic acid; VA, Vanillic acid; PH, plant height; LA, leaf area; SDW, Shoot dry weight; RDW, Root dry weight; RV, Root vitality; RSA, Root surface area; Yid, Yield; FAA, Free amino acid content; VC, Vitamin C content; SS, Soluble sugar content; TA, Titration acid content.

Under both vermicompost treatments, plant growth and fruit yield in strawberries, along with all quality parameters except for organic acids, showed positive correlations with the bacterial count and nutrient content of the substrates. Conversely, these parameters were significantly negatively correlated with fungal count and phenolic acid levels in the substrates (**Figure 8**). In both substrates, fruit yield exhibited strong significant correlations with bacterial and fungal counts, available nitrogen content, plant height, aboveground dry weight, root volume, vitamin C content, soluble sugar content, and the sugar–acid ratio (**Figure 8**).

### 3.7 The comprehensive impact of vermicompost treatments on strawberry substrate, plant growth, and fruit quality

The membership function analysis of component contents indicated that the CV treatment in the 0-year substrate had the highest ranking (**Table 3**), while the CK treatment in the 2-year substrate had the lowest. The ranking of CV treatment in the 2-year substrate was higher than that of SV treatment, both of which were higher than CK in the 0-year substrate. Across both the 0-year and 2-year substrates, the CV treatment consistently ranked higher than the SV treatment.

**Figure 9:**
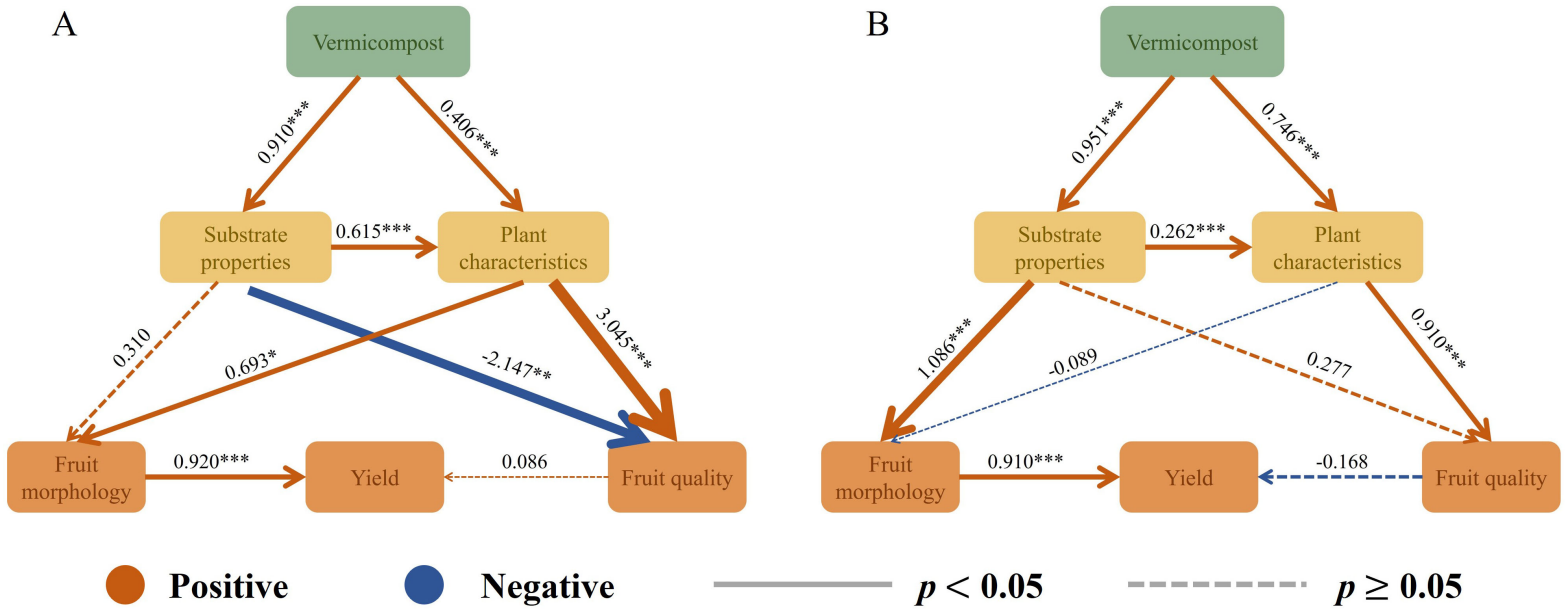
The structural equation model (SEM) shows the effects of vermicompost on substrate properties, plant indicators, fruit morphology, fruit quality, and yield. Sequences **(A, B)** represent substrates with 0 and 2 years of cropping, respectively. Yellow lines and blue lines indicate positive and negative coefficients, respectively. Solid lines denotesignificant effects. (*p*·< 0.05), and dashed lines indicate no significant effects (*p* ≥ 0.05). The arrows represent standardized path coefficients, and their width indicates the strength of the coefficients.

The SEM analysis further indicated the relationship between substrate properties, plant growth, fruit morphological quality, and yield with the application of vermicompost (**Figure 9**). Vermicompost directly affected substrate properties and plant growth, which in turn affected fruit morphology and ultimately fruit yield. Vermicompost treatments also had an indirect impact on fruit quality by altering substrate properties, potentially leading to negative effects (**Figure 9A**).

## 4 Discussion

### 4.1 Effects of vermicompost treatments on substrate properties

Vermicompost is rich in plant-available nutrients, including nitrates, phosphates, exchangeable calcium, and soluble potassium. In this study, vermicompost application significantly influenced the contents of total phosphorus (**Figure 1D**), total potassium (**Figure 1E**), alkali-hydrolyzable nitrogen (**Figure 1F**), and available phosphorus (**Figure 1G**) in the substrates across two cropping durations. Vermicompost also has a diverse microbial population (Naseer and Shahid, 2018), which is a result of the polysaccharides, proteins, and other nitrogenous compounds secreted by earthworms during compost formation. These secretions create resource heterogeneity, which ultimately improve microbial diversity in the vermicompost (Hussain et al., 2016b; a). In the present study, the applied vermicompost promoted bacterial (**Figure 4A**) and actinomycete (**Figure 4C**) survival in both substrates while resulting in a reduction of the fungal population (**Figure 4B**). These results are consistent with previous studies (Akinnuoye-Adelabu et al., 2019; Wang et al., 2021a). Lu et al. (2021) reported that vermicompost application significantly reduces fungal populations, thereby effectively decreasing the incidence of lily wilt, alleviating continuous cropping obstacles, and promoting stable and increased yields. Cattle manure vermicompost was most effective in enhancing both bacterial and fungal populations in the substrates (**Figures 4A, B**), while sludge-based vermicompost exhibited a stronger impact on actinomycete growth (**Figure 4C**). This variation may be linked to the differing origins of the two types of vermicompost, each containing distinct microbial communities.

Degradation of the soil microecological environment is a major obstacle to continuous cropping (Yang et al., 2016). Long-term continuous cropping triggers a shift in the soil microbial community structure from “bacterial-dominated” to “fungal-dominated,” thus disrupting the microbial balance (She et al., 2017; Pan et al., 2019). Toxic substance accumulation is another major factor leading to continuous cropping challenges (Xiong et al., 2015). Phenolic acid accumulation is a primary factor contributing to continuous cropping issues (Xie et al., 2017). Phenolic acids, such as p-hydroxybenzoic acid, vanillic acid, p-coumaric acid, and ferulic acid, are often present in soil and inhibit crop growth (Bai et al., 2019). In this study, the addition of vermicompost reduced the levels of ferulic acid (**Figure 2B**) and vanillic acid (**Figure 2E**), which is consistent with prior study (Pan et al., 2019). The incorporation of vermicompost mitigated the allelopathic effects of phenolic acids in substrates subjected to two years of continuous cropping. This phenomenon can be attributed to two primary factors: first, vermicompost effectively reduces the phenolic acid content in the substrate due to the strong adsorption properties of its humic substances and other components. Second, the high microbial content present in vermicompost may facilitate the degradation of phenolic acids by specific microorganisms (Sun et al., 2020). Moreover, regardless of the cropping history duration (0- or 2 years), the cattle manure vermicompost. This study showed that the vermicompost application effectively enhanced the chemical properties of the substrate (**Figure 1**) and increased the activities of the substrate enzymes, including sucrase (**Figure 3A**), substrate catalase (**Figure 3B**), urease (**Figure 3C**), polyphenol oxidase (**Figure 3D**), acid phosphatase (**Figure 3E**), and alkaline phosphatase (**Figure 3F**). These findings are consistent with previous research showing that vermicompost application was found to significantly improve soil enzyme activity in honeysuckle and promote plant growth (Guo et al., 2023).

### 4.2 Effects of vermicompost application on plant growth

In this study, vermicompost treatments led to varying degrees of positive effects on plant height (**Figure 5A**), leaf area (**Figure 5C**), root and stem dry weight (**Figures 6A, B**), and root growth (**Supplementary Figure S4**) across different planting years and growth stages. Notably, the effect of vermicompost was the most pronounced because it is enriched with diverse microorganisms and plant hormones (He et al., 2020; Lin et al., 2021). These hormones are believed to promote root development, enhance soil nutrient uptake, promote plant growth, and boost biomass accumulation (Haghighi et al., 2016; Esteves et al., 2020).

In the present study, the application of vermicompost also enhanced the leaf chlorophyll content (**Figure 5**), photosynthesis rate (**Supplementary Figure S2**), and leaf nutrient content (**Supplementary Figure S3**) across the two planting durations. The treatments augmented the ability of plant to absorb mineral elements (Najar et al., 2015), increased leaf nutrient content, and supplied adequate nitrogen for synthesizing chlorophyll, thereby increasing the leaf chlorophyll content and photosynthesis. Chlorophyll-generated photosynthetic products supply adequate energy for leaf growth and metabolism, thus facilitating the maintenance of normal physiological functions and fostering overall plant growth (Hosseinzadeh et al., 2016; Zuo et al., 2018). Consequently, leaf enzyme activity further improved (**Figures 6C–F**), which aligns with previous results (Subhasish et al., 2017). Moreover, this study revealed that vermicompost treatment improves plant growth by modifying substrate properties. The SEM results indicated that vermicompost and substrate properties across both 0-year and 2-year planting histories positively influenced the growth of strawberry plants (**Figure 9**). This finding aligns with previous studies that have reported the influence of soil nutrient content, a critical substrate property, influenced cucumber growth (Zantis et al., 2024).

### 4.3 Effects of vermicompost treatments on strawberry yield and quality

This study showed that irrespective of planting duration, vermicompost treatment significantly increased single fruit weight and the diameter and yield of strawberries (**Table 4**). Notably, the application of cattle manure vermicompost exhibited the most pronounced effect, consistent with findings from previous studies (Yohannes et al., 2023). Vermicompost is known to enhance crop yield. During decomposition, vermicompost improves the soil structure, facilitates dynamic interactions at the root–soil interface, and enhances microbial activity, these factors collectively promote plant growth and increase fruit accumulation in cucumbers (Maji et al., 2017). The SEM results indicated that the improved properties of substrates without and with 2-year planting history directly or indirectly affected fruit morphology, which positively influenced the yield (**Figure 9**). The substantial increase in soil microbial biomass and nutrient content after vermicompost addition may have partly led to improvements in crop growth and increased fruit yield. This enhancement in soil quality likely resulted in elevated levels of hormones and humic acids in the treated crops (Sudip et al., 2023). Our findings confirmed that microbial count and nutrient content in the substrate increased after vermicompost application (**Figures 1**, **4**).

**Table 4:** Effects of vermicompost on strawberry fruit morphology and yield in different periods.

Cropping years	Period	Treatment	Weight of single fruit (g)	Transverse diameter of fruit (mm)	Fruit longitudinal diameter (mm)	Yield per plant (g)	Yield (kg/ha)
0	P2	CK	21.78 ± 1.27 b	33.05 ± 2.60 b	42.41 ± 2.65 b	—	—
		SV	27.51 ± 1.69 a	38.30 ± 2.21 a	51.73 ± 4.01 a		
		CV	28.72 ± 1.63 a	41.21 ± 0.62 a	53.24 ± 2.81 a		
	P3	CK	18.15 ± 1.36 b	31.84 ± 0.58 b	43.82 ± 0.88 b	390.68 ± 12.17 b	34509.59
		SV	21.85 ± 1.09 a	35.43 ± 0.67 a	47.46 ± 3.28 a	443.36 ± 23.01 a	
		CV	22.09 ± 0.95 a	35.29 ± 1.40 a	49.47 ± 1.38 a	462.13 ± 22.23 a	40821.31
2	P2	CK	19.46 ± 1.04 c	32.35 ± 2.10 b	41.96 ± 2.07 b	—	—
		SV	24.01 ± 1.16 b	38.36 ± 1.67 a	50.42 ± 2.29 a		
		CV	25.97 ± 0.95 a	39.34 ± 0.45 a	52.11 ± 2.17 a		
	P3	CK	17.19 ± 1.23 b	31.43 ± 1.50 b	43.06 ± 1.09 b	360.66 ± 12.86 b	31857.95
		SV	19.32 ± 1.33 ab	33.62 ± 0.96 a	46.02 ± 1.42 a	419.6 ± 22.31 a	37064.36
		CV	20.88 ± 1.50 a	34.25 ± 0.96 a	45.87 ± 1.72 a	431.49 ± 7.97 a	38114.73

The letters indicate the significant difference of the influence of different vermicompost on the index, and the significance level is *p* < 0.05. P2 and P3 represent peak fruiting period and the senescence phase respectively. CK, Control; SV, sludge vermicompost; CV, cattle manure vermicompost. The same below.

Continuous cropping causes a decline in fruit quality. Interestingly, our findings showed that vermicompost application significantly increased the soluble solid content (**Figure 7A**), free amino acid content (**Figure 7B**), ascorbic acid content (**Figure 7C**), and soluble sugar content (**Figure 7D**) of fruits; decreased fruit titratable acid content (**Figure 7E**); and elevated the fruit sugar–acid ratio (**Figure 7F**), thereby augmenting fruit flavor. In cucumbers, across different continuous cropping years, vermicompost treatment was reported to significantly affect yield, increase vitamin C and soluble sugar contents, and reduce fruit acidity and nitrate levels compared with the control (Wang et al., 2021a). The SEM results demonstrated that across both of planting histories (0- and 2-year), fruit quality was positively influenced by plant indicators (**Figure 9**).In the present study, vermicompost treatments increased nutrient content of the substrate, thereby augmenting the leaf nutrient content. The enhancement in strawberry fruit quality is hypothesized to be related to the increased potassium content. Moreover, vermicompost contains various plant growth hormones and essential amino acids, such as glutamic acid and glycine. These components play a vital role in soluble sugar metabolism through plant roots, thus boosting the soluble sugar content of fruits and significantly enhancing their quality (Scaglia et al., 2016).

The results indicated that, irrespective of the substrate type, the cattle manure vermicompost consistently yielded highest. On analyzing the properties of the two vermicompost types (**Supplementary Table S1**), we found that the aforementioned result may be attributable to the higher electrical conductivity of the sludge vermicompost compared to the cattle manure vermicompost, as an elevation in ion concentrations can influence plant growth and potentially lead to plant death (Lazcano et al., 2009). Moreover, in the 2-year continuous cropping substrate, both SV and CV treatments ranked higher than the CK treatment for the 0-year substrate, underscoring the effectiveness of vermicompost treatment in mitigating continuous cropping obstacles.

## 5 Conclusion

This study showed that vermicompost treatments significantly improved the quality of strawberry substrates, increasing the nutrient levels and reducing phenolic acid content of the substrates, irrespective of planting years, compared with the CK treatment. Consequently, these treatments augmented the growth and physiological indices of both aboveground and underground plant parts, promoted photosynthesis, and facilitated greater nutrient accumulation, contributing to improved fruit yield and quality. According to the SEM analysis, vermicompost treatments significantly influenced substrate properties, plant indicators, and fruit morphology, leading to enhanced fruit yield and quality. The membership function results demonstrated the superiority of the cattle manure vermicompost to the sludge vermicompost. Notably, the cattle manure vermicompost added to the 2-year substrate ranked higher in the membership function than the CK treatment of the 0-year planting substrate. These findings indicate that the integration of vermicompost is beneficial for plant growth in continuous cropping systems. Consequently, this study advocates for the use of cattle manure vermicompost as a best practice for improving the quality and yield of strawberry fruits.

## Data Availability

The original contributions presented in the study are included in the article/**Supplementary Material**. Further inquiries can be directed to the corresponding authors.
