# Supplementary material for: Effects of cattle manure and sludge vermicompost on nutrient dynamics and yield in strawberry cultivation with distinct continuous cropping histories in a greenhouse
Source: Front Plant Sci. 2025 Jan 6;15:1514675. doi: 10.3389/fpls.2024.1514675 (PMC11743566; doi:10.3389/fpls.2024.1514675)
Supplement: Supplementary file 1 [file DataSheet1.docx]

**Supporting materials**

Table S1 Basic properties of two kinds of vermicompost

| Traits | Sludge vermicompost | Cattle manure vermicompost |
| --- | --- | --- |
| Bulk density (g/cm^3^) | 0.54 | 0.75 |
| Total porosity (%) | 64.70 | 55.72 |
| Aeration porosity (%) | 4.28 | 5.90 |
| Water-holding porosity (%) | 60.41 | 49.82 |
| Gas water ratio | 0.07 | 0.12 |
| pH | 6.93 | 7.07 |
| Electrical conductivity (mS/cm) | 1.38 | 1.27 |
| Total nitrogen content (g/kg) | 16.67 | 8.51 |
| Total phosphorus content (g/kg) | 11.41 | 6.36 |
| Total potassium content (g/kg) | 10.17 | 12.17 |
| Hydrolyzed nitrogen content (g/kg) | 1.51 | 0.91 |
| Available phosphorus content (g/kg) | 0.35 | 0.38 |
| Available potassium content (g/kg) | 1.77 | 0.83 |
| Organic matter content (g/kg) | 262.14 | 192.50 |

Table S2 Content of heavy metals in two kinds of vermicompost

| Traits | Sludge vermicompost | Cattle manure vermicompost |
| --- | --- | --- |
| Arsenic content (mg/kg) | 12.96 | 7.87 |
| Cadmium content (mg/kg) | 0.52 | 0.25 |
| Chromium content (mg/kg) | 47.65 | 61.73 |
| Mercury content (mg/kg) | 0.83 | 0.28 |
| Lead content (mg/kg) | 78.51 | 39.04 |


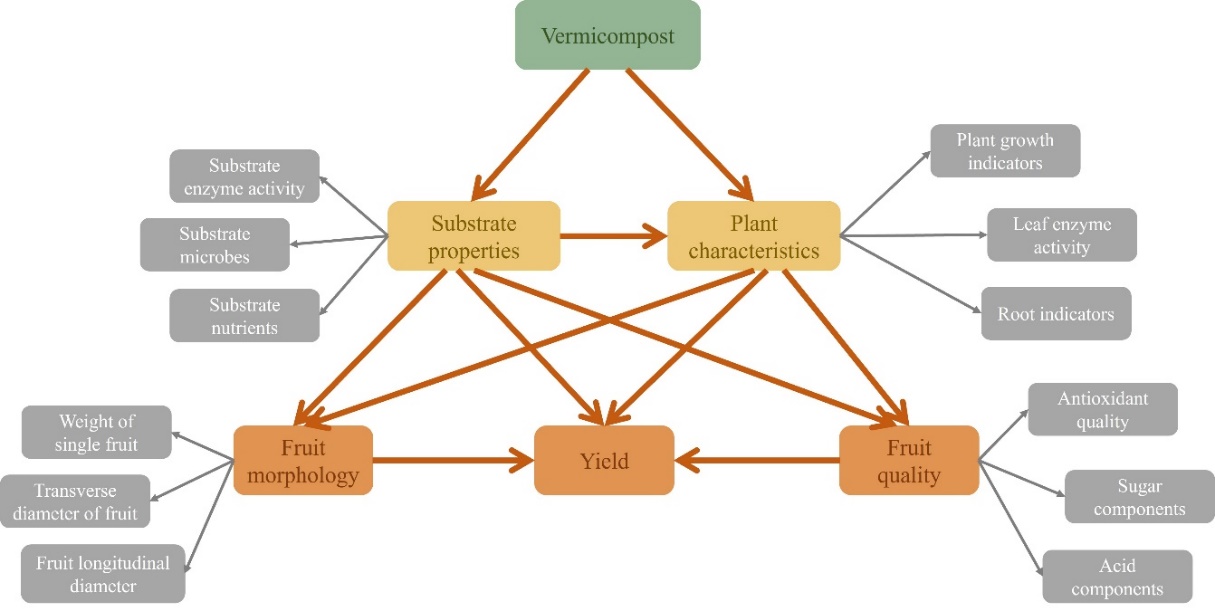


Fig. S1 Hypothetical conceptual model


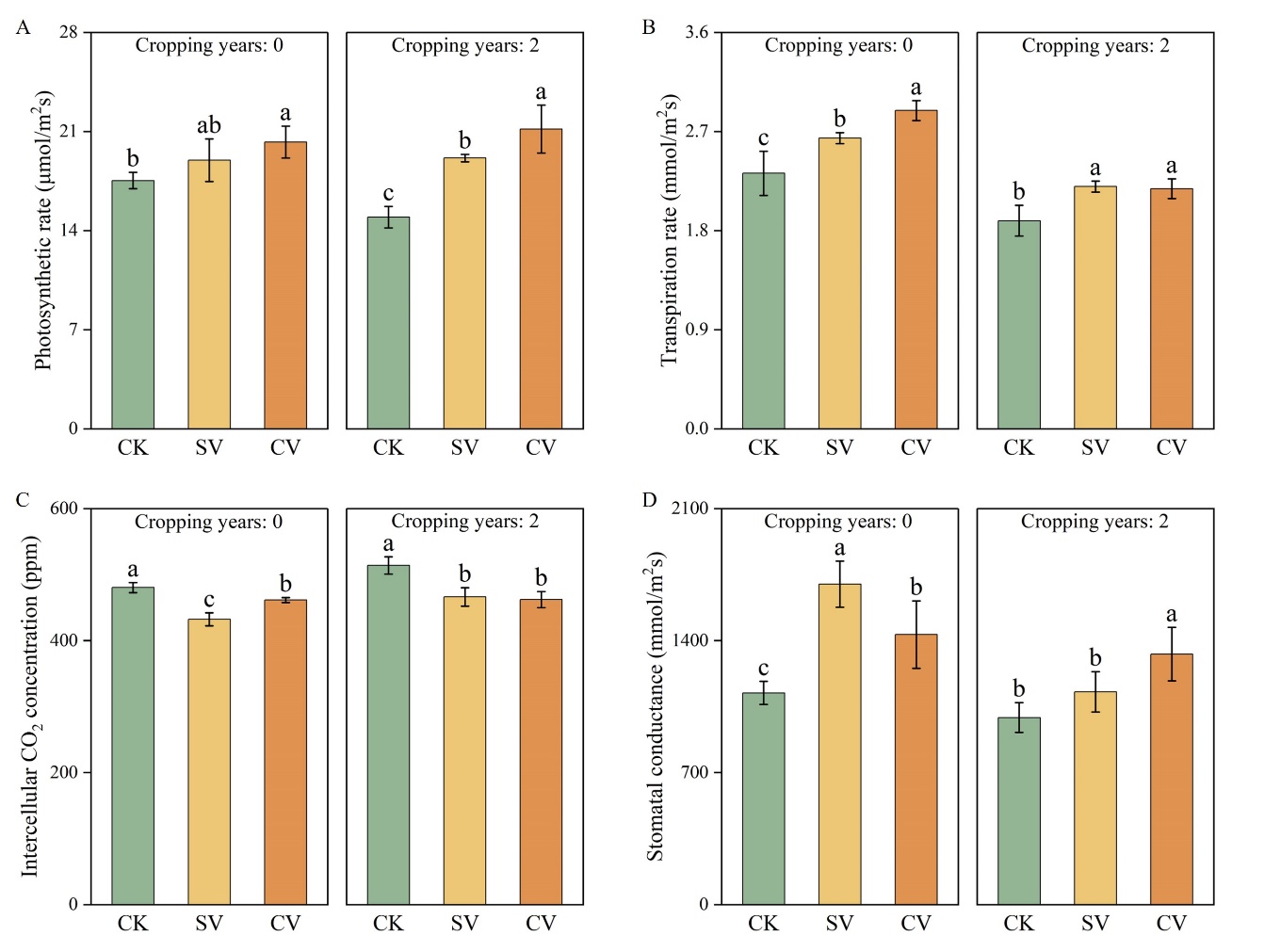


Fig. S2 Effects of vermicompost on photosynthetic rate (A), transpiration rate (B), intercellular CO_2_ concentration (C) and stomatal conductance (D)at the peak fruiting period. The letters indicate the significant difference of the influence of different vermicompost on the index, and the significance level is *p* < 0.05. Abbreviations for treatment: Control (CK), sludge vermicompost (SV) and cattle manure vermicompost (CV).

**
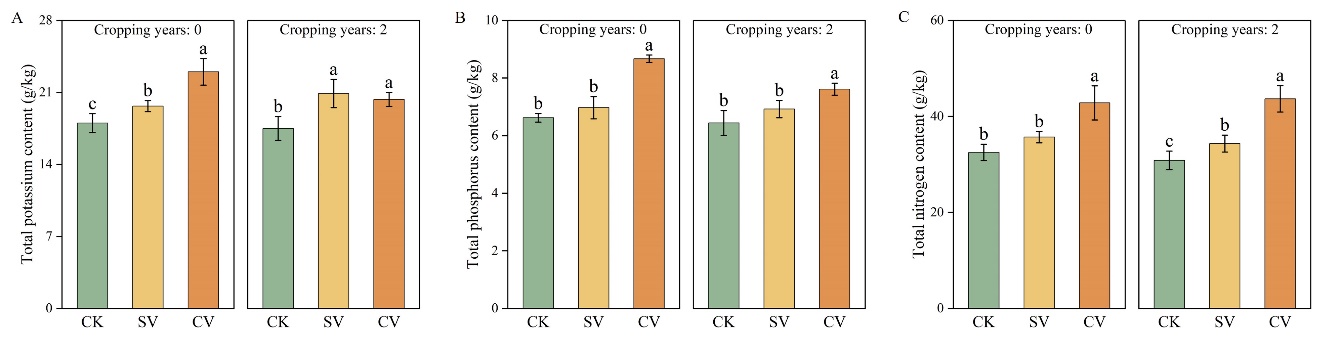
**

Fig. S3 Effects of vermicompost on total potassium content (A), total phosphorus content (B) and total nitrogen content (C)

at full bearing period. The letters indicate the significant difference of the influence of different vermicompost on the index, and the significance level is *p* < 0.05. Abbreviations for treatment: Control (CK), sludge vermicompost (SV) and cattle manure vermicompost (CV).


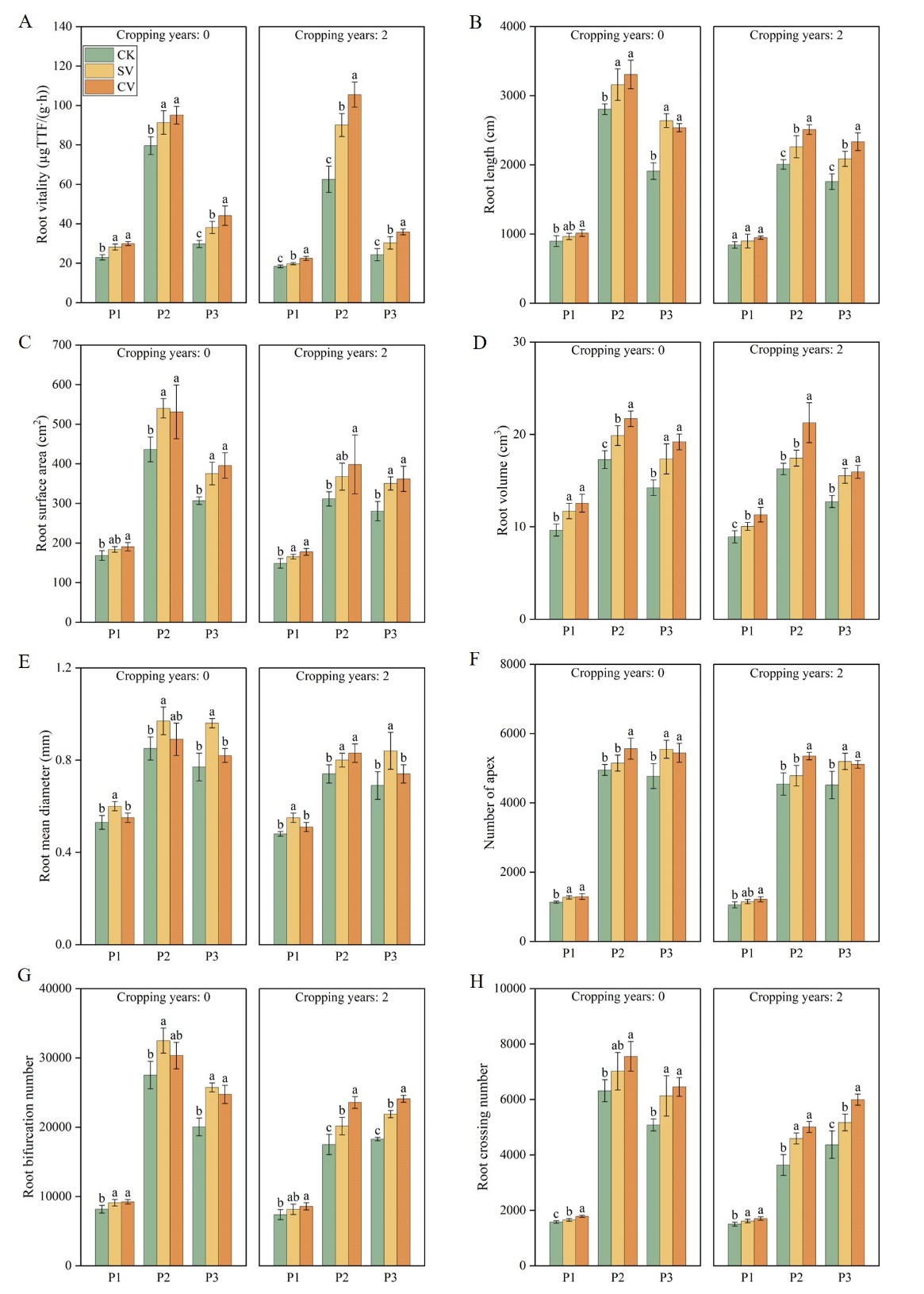


Fig. S4 Effects of vermicompost on root vitality (A), root length (B), root surface area (C), root volume (D), root mean diameter (E), number of apex (F), root bifurcation number (G) and root crossing number (H) in different periods. Letters indicate significant differences in the effects of different vermicompost treatments on this indicator at different time periods, with a significance level of *p* < 0.05. P1, P2 and P3 represent flowering period, peak fruiting period and the senescence period respectively. Abbreviations for treatment: Control (CK), sludge vermicompost (SV) and cattle manure vermicompost (CV).

**
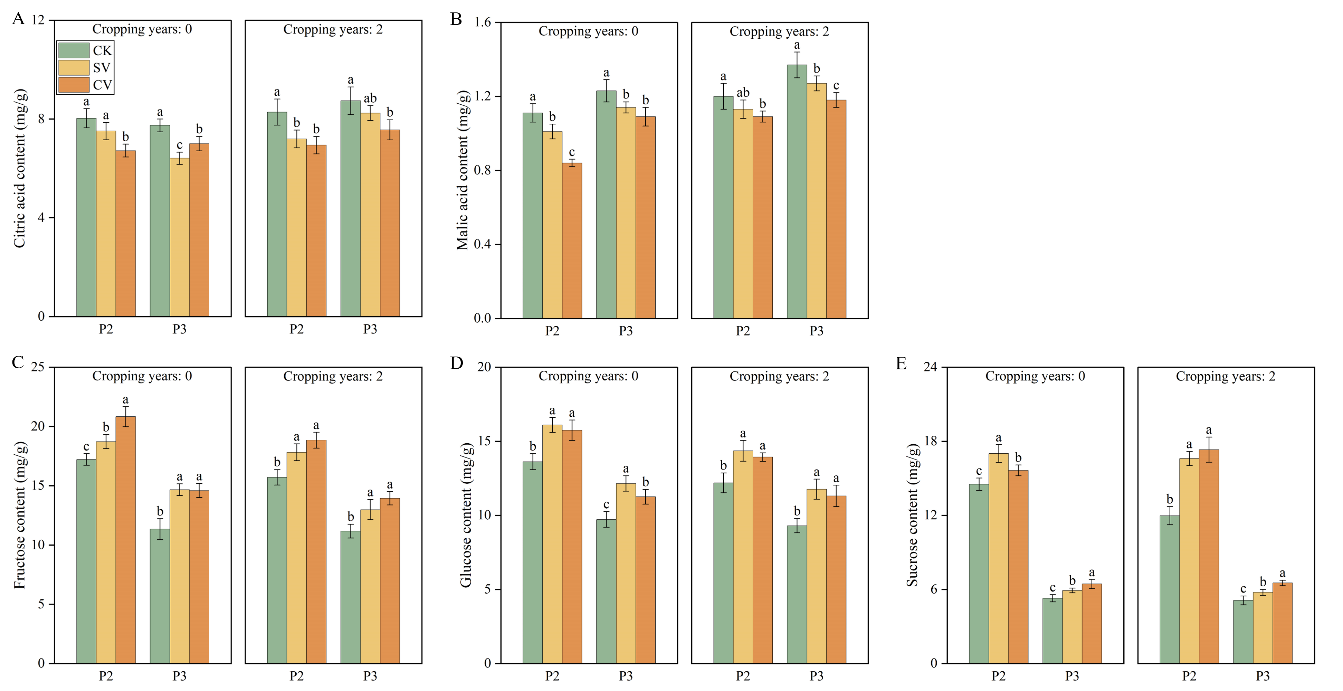
**

Fig. S5 Effects of vermicompost on citric acid content (A), malic acid content (B), fructose content (C), glucose content (D), sucrose content (E) in different periods. Letters indicate significant differences in the effects of different vermicompost treatments on this indicator at different time periods, with a significance level of *p* < 0.05. P2 and P3 represent peak fruiting period and the senescence period respectively. Abbreviations for treatment: Control (CK), sludge vermicompost (SV) and cattle manure vermicompost (CV).
